# Optimization of Three-Phase Hybrid Stepper Motors for Noise Reduction

**DOI:** 10.3390/s22010356

**Published:** 2022-01-04

**Authors:** Zhen Peng, Chao Bi, Lingli Fang, Longfei Xiao

**Affiliations:** 1 Department of Control Science and Engineering, University of Shanghai for Science and Technology, Shanghai 200093, China; pengzhen324@outlook.com; 2 Department of Electrical Engineering, University of Shanghai for Science and Technology, Shanghai 200093, China; 182540366@st.usst.edu.cn (L.F.); 182540372@st.usst.edu.cn (L.X.)

**Keywords:** optimization, torque ripple, acoustic noise, total harmonic distortion (THD), finite element, Taguchi method

## Abstract

For the optimization of three-phase hybrid stepper motors with complex electromagnetic structures, an optimization method is presented in this paper. The method is a combination of 3D-FEM and the Taguchi optimization method intended to reduce the dependence on FEM results during the optimization calculation. In this paper, the optimization method is used in the optimization of the tooth shape of the three-phase hybrid stepper motor, and the objective is to reduce the noise caused by harmonics in the “torque-angle characteristic” of the motor. It is clear that traditional optimization methods make it very difficult to carry out such an optimization calculation as a large number of finite element calculations have to be used in the optimization process, and the required computation time is extremely long. Using the optimization method presented in the paper, the optimization becomes feasible because the number of finite element calculations is greatly reduced and the computation time is thus greatly reduced. In order to check the effectiveness of the optimization, the waterfall diagram for noise analysis and its application to check torque ripple are also presented in the paper. Both simulation and test results show that the optimized structure can significantly reduce the motor noise caused by torque ripple. Therefore, the optimization method proposed in this paper can be an effective tool for the optimal design of high-performance motors, including stepper motors.

## 1. Introduction

Hybrid stepper motors (HSMs) are widely used in control systems because of their high positioning accuracy, compact size, and lower operation noise [1]. Among various HSMs, the 3P-HSM has more advantages, e.g., higher torque and fewer MOSFETs in the control system, and thus, will dominate the HSMs market with the increase in market requirements in terms of motor performance and cost [1,2]. 

The optimization of HSMs is very difficult because of the complexity of the motor structure, the small air gap of the motor, and the presence of both axial and radial magnetic fields [3,4,5,6]. The three-dimensional finite element method (FE method, FEM) is an effective tool for the analysis of such motors [4], but they are extremely computationally intensive and require very long computation times, making them ineffective for the optimization of HSMs.

The 3P-HSM has a multi magnetic-pole structure and the most common structures include six-poles and 12-poles. Based on the unique electromagnetic (EM) structure of 3P-HSM, the reduction in motor torque ripple and noise has always been a research hotspot [5]. The reduction is clearly related with the optimization of the motor design [5,6]. It is clear that traditional optimization methods make it very difficult to carry out such an optimization calculation as a large number of FE calculations have to be used in the optimization process, and the required computation time is so long that optimization using FE calculations becomes infeasible [7]. This paper presents the Taguchi method for the optimization of the EM structure of HSMs. The aim of the optimization is to reduce the torque ripple and operation noise of the motor [8]. The Taguchi algorithm was proposed by Gen’chi Taguchi in the 1950s. Through local optimization, the Taguchi algorithm can establish the lowest FE model using the smallest number of tests, and use discrete data to find the best combination, which greatly reduces the time cost [9,10,11,12].

The sources of acoustic noise in the motor operation can be classified into three categories: mechanical, aerodynamic, and EM noise [5]. In the 3P-HSM, the EM acoustic noise is mainly related to the drive circuit. The drive circuit noise is induced by the drive current with high-order harmonics. The drive current generates EM torque ripples, and then induces the vibration and acoustic noise in the motor’s operation [7]. This noise type is not discussed here. This paper focuses on the EM noise caused by cogging torque ripples. Optimizing the motor tooth shape to reduce the THD is an effective method in terms of reducing motor cogging torque and improving motor noise.

In order to minimize the THD, it is necessary to analyze and optimize the EM structure of 3P-HSM. Using a 3D FEM model, it is very difficult and time-consuming to carry out an EM analysis even for the 3P-HSM, let alone to optimize the motor [13,14,15]. Taking the computation in the authors’ research on 3P-HSM as an example, it is known that one 3D FE mode needs about 1 million elements and 10 million nodes. For every “Torque-angle characteristic” curve, the torque for 30 rotor positions was calculated, and every FE computation needed about 2 h. Considering that four parameters needed to be optimized when using the FEM directly for the optimization, it is clear that it is not practical to use FEM directly for optimization calculations.

**Figure 1:**
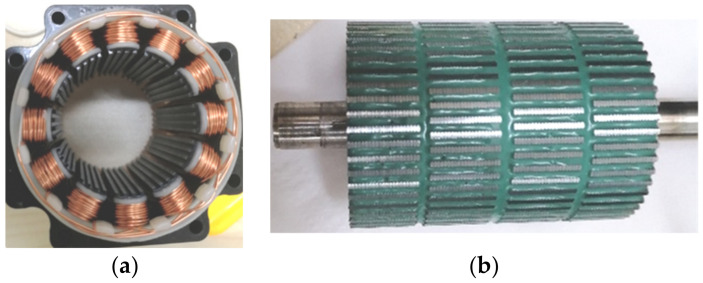
The practical product for the initial 3P-HSM model: (**a**) 3P-HSM with 12 slots; (**b**) rotor structure.

In this paper, a method that combines the Taguchi method with FEM is introduced to optimize the tooth structure. This method can significantly reduce the results obtained via the FEM. A practical product for 3P-HSM is selected as the prototype for optimal design, which is shown in Figure 1. The optimization is aimed at reducing the THD of the holding torque. Furthermore, the testing results of the optimal THD-min 3P-HSM confirm the effectiveness of the optimization method.

## 2. Torque-Angle Characteristics

**Figure 2:**
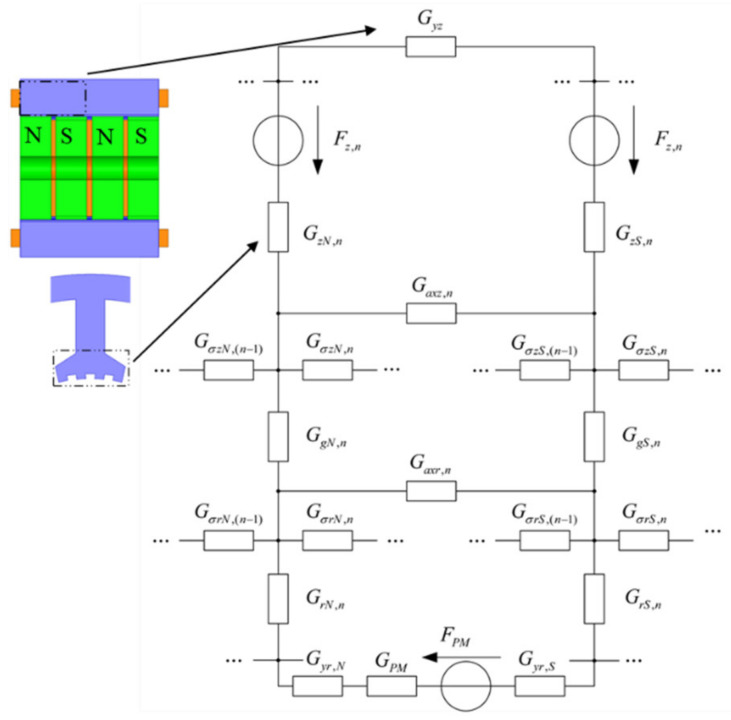
The equivalent magnetic circuit of the stator part of the HSM.

The torque-angle characteristics reflect the ability of the stepper motor to produce EM torque. Many researchers and engineers use the magnetic circuit method for calculations relating to motor design [16]. Unlike the structure of other synchronous motors, the HSM has a more complex magnetic circuit, which contains both radial and axial magnetic paths. Figure 2 shows the equivalent magnetic circuit of the motor. 

The energy of the HSM is mainly provided by the stator winding and permanent magnets. The energy *dW_e,n_* provided by the nth stator winding is as follows:

(1) $$dW_{e,n}=i_{n}N_{n}d(\phi_{z,n})=F_{z,n}d(\phi_{zN,n}+\phi_{zS,n})$$

where *i_n_* is the stator winding’s current, *N_n_* represents the stator winding’s turns, *F_z,n_* is the stator winding’s magnetic potential, *ϕ_z,n_* is the magnetic flux of the n-th pole stator’s teeth. Due to the adjacent rotor teeth differing by 1/2 tooth pitch, the magnetic field is divided into the N-side and the S-side. *ϕ_zN,n_* and *ϕ_zS,n_* are the tooth magnetic flux on the N-side and S-side, respectively.

The energy *dW_e,PM_* provided by the permanent magnet is shown [11]:

(2) $$dW_{e,PM}=F_{PM}\phi_{PM}$$

where *F_PM_* and *ϕ_PM_* are the magnetic potential and the magnetic flux of the permanent magnet, respectively.

The magnetic energy *dW_mag_* and the output mechanical energy *dW_mech_* can be expressed [12]:

(3) $$dW_{mag}=\frac{1}{2}d\left(\frac{\phi^{2}}{G}\right)$$

where *G* is the magnetic permeance and *ϕ* is the magnetic flux of each part.

(4) $$dW_{mech}=Td\theta$$

where *T* is the torque and *θ* is the rotor rotation angle.

Considering the energy conservation, the torque can be obtained:

(5) $$dW_{e}=dW_{mag}+dW_{mech}$$

(6) $$\begin{array}{cc} T & =\frac{d}{d\theta }\left(W_{e,n}+W_{e,PM}\right)-\frac{d}{d\theta }\left(W_{mag}\right)\\ & =\frac{d}{d\theta }\left\{\sum_{n=1}^{12}\left[F_{z,n}(\phi_{zN,n}+\phi_{zS,n})\right]+F_{PM}\phi_{PM}\right\}\\ & -\frac{1}{2}\frac{d}{d\theta }\left[\sum_{n=1}^{12}(\frac{\phi_{yz,n}^{2}}{G_{yz,n}}+\frac{\phi_{zN,n}^{2}}{G_{zN,n}}+\frac{\phi_{zS,n}^{2}}{G_{zS,n}}+\frac{\phi_{axz,n}^{2}}{G_{axz,n}}+\frac{\phi_{\sigma zN,n}^{2}}{G_{\sigma zN,n}}+\frac{\phi_{\sigma zS,n}^{2}}{G_{\sigma zS,n}}+\frac{\phi_{gN,n}^{2}}{G_{gN,n}}+\frac{\phi_{gS,n}^{2}}{G_{gS,n}}\right.\\ & \left.+\frac{\phi_{axr,n}^{2}}{G_{axr,n}}+\frac{\phi_{\sigma rN,n}^{2}}{G_{\sigma rN,n}}+\frac{\phi_{\sigma rS,n}^{2}}{G_{\sigma rS,n}}+\frac{\phi_{rN,n}^{2}}{G_{rN,n}}+\frac{\phi_{rS,n}^{2}}{G_{rS,n}}+\frac{\phi_{yr,N}^{2}}{G_{yr,N}}+\frac{\phi_{yr,S}^{2}}{G_{yr,S}})\right]\\ & -\frac{1}{2}\frac{d}{d\theta }\left(\frac{\phi_{PM}^{2}}{G_{PM}}\right) \end{array}$$

where *ϕ_vz_* is the stator’s axial yoke’s magnetic flux, and *ϕ_axz_* and *ϕ_axr_* represent the stator and rotor’s axial tooth’s leakage magnetic flux, respectively. *ϕ_δzN,n_* and *ϕ_δzS,n_* are the N-side and S-side’s stator radial inter-tooth’s leakage flux, *ϕ_gN,n_* and *ϕ_gN,n_* are the N-side and S-side’s air gap’s magnetic flux, *ϕ_δrN,n_* and *ϕ_δrS,n_* are the N-side and S-side’s rotor’s radial inter-tooth’s leakage flux, *ϕ_rN,n_* and *ϕ_rS,n_* are the N-side and S-side’s rotor’s radial inter-tooth’s magnetic flux, and *ϕ_vr,N_* and *ϕ_vr,S_* are the N-side and S-side’s rotor’s axial yoke’s magnetic flux.

In the actual optimization process, a slight change in the tooth structure has a great impact on the air gap’s magnetic field density. At the same time, the local magnetic saturation of HSMs must be considered. Therefore, the magnetic circuit method and FEM are often combined in the design of motors to improve the accuracy of the calculation results with regard to the torque-angle characteristics.

## 3. FEM Model of 3P-HSM

Due to its very complex EM structure and the large number of non-linear materials used in the motor, combined with the very small step angle, conventional methods, such as the equivalent magnetic circuit method, are unable to obtain highly accurate results for the analysis and calculation of HSMs. In recent years, designers have attempted to utilize 3D models to calculate the characteristic of motors [17,18]. Compared with other methods, FEM is better suited to the analysis and design of HSMs.

**Figure 3:**
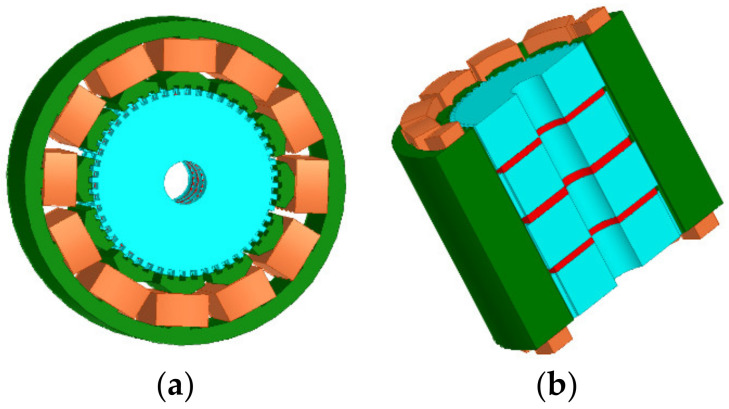
The 3D model of initial 3P-HSM: (**a**) 3D model of the 3P-HSM with 12 slots; (**b**) axial sectional view of the motor.

**Figure 4:**
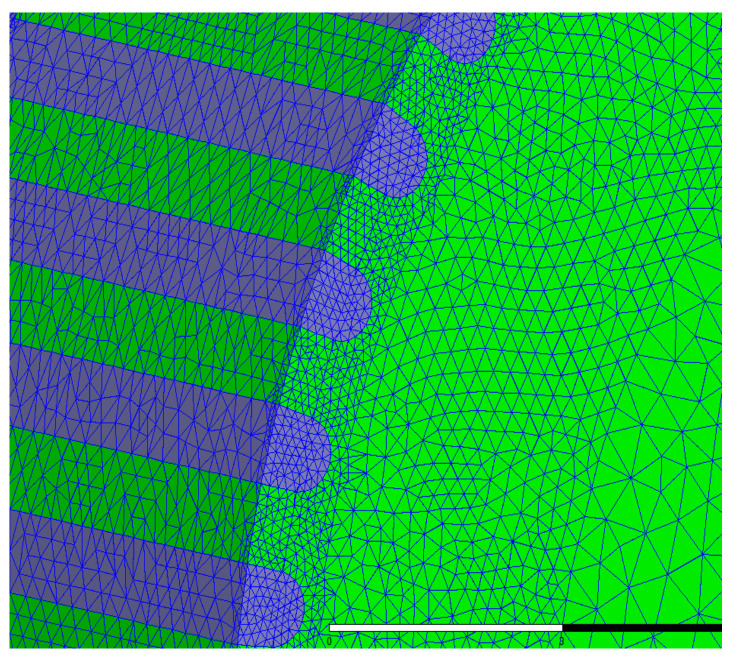
The FEM mesh around the rotor teeth of the motor.

**Figure 5:**
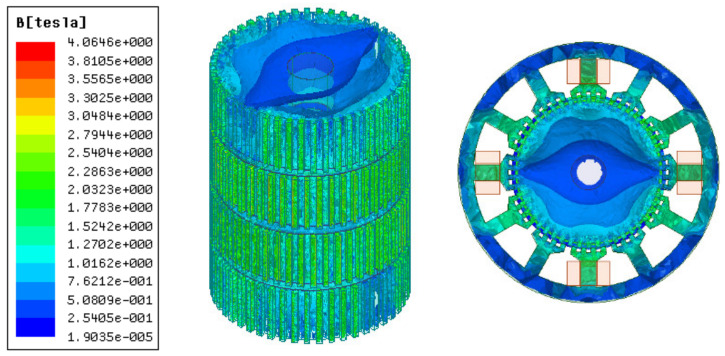
The magnetic flux density of single-phase winding energized with direct current.

Figure 3 and Figure 4 illustrate 3D models of the initial 3P-HSM and FEM mesh around the rotor teeth of the motor. The number of turns of the stator winding was 62. The magnetic density of the motor when 1.9A DC was applied to one-phase winding is shown in Figure 5. For HSMs, the tooth layer structure, which directly affects the tooth shape, is one of the main factors that determine the performance of the motor. In the calculations, high density meshes were used in the areas close to the iron small teeth; this, of course, significantly increased the computation time in the 3D-FEM calculation, in line with the findings given in [19]. In order to effectively utilize the results obtained from the 3D-FEM, the optimization of HSMs relies on the Taguchi optimization method, and this was also used in the authors’ research. The process will be presented in detail in the next section.

The main calculated parameters of the 3P-HSMs are listed in Table 1 and Table 2. For HSMs, the tooth geometry is closely related with motor performance, and thus, was naturally selected for optimization.

**Figure 6:**
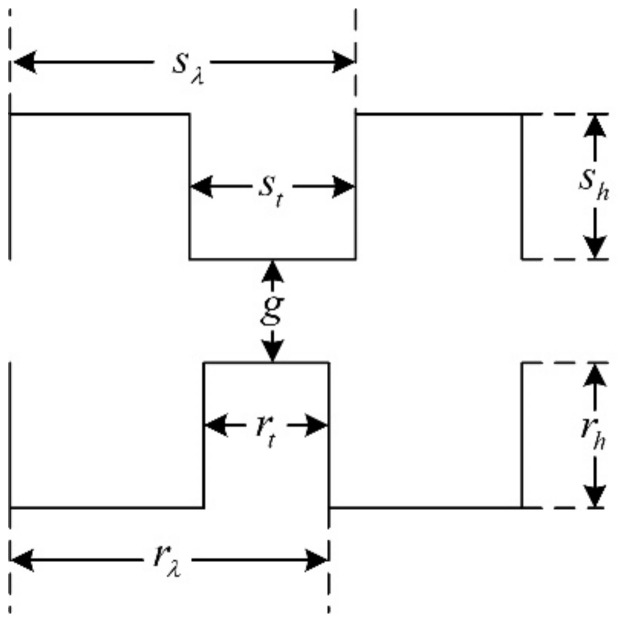
The rectangular teeth.

**Figure 7:**
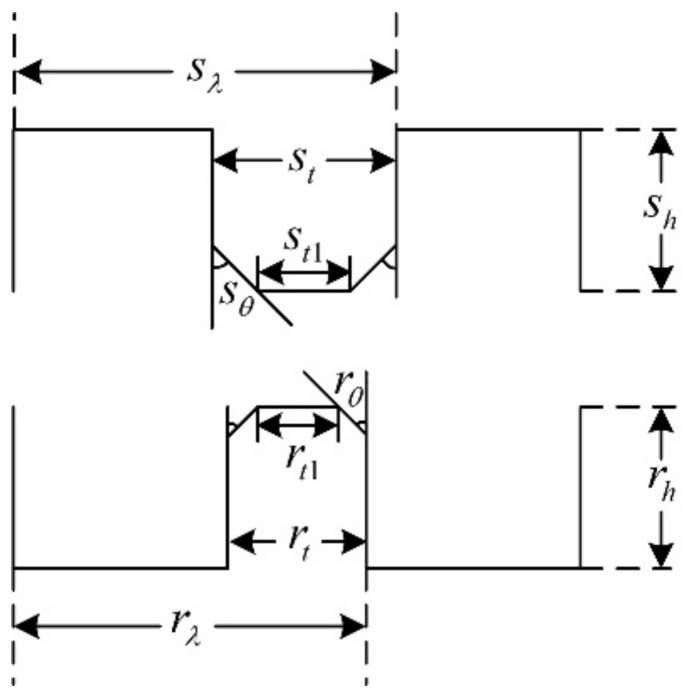
The trapezoidal teeth.

In the past decades, researchers put forward different opinions about the tooth shape, such as it being rectangular, triangular, circular, and so on [20,21,22]. Among them, the rectangular tooth shape shown in Figure 6 has been widely used in many products. However, according to practical experience, the trapezoidal tooth shape, as shown in Figure 7, is much better than the rectangular one, because this tooth profile contains more shape parameters and allows more degrees of freedom for optimization, making it easier to achieve the required motor performance.

## 4. Optimization of 3P-HSM

In order to efficiently optimize HSMs with complex EM structures, an optimization approach combining FEM and the Taguchi optimization method was developed.

### 4.1. Taguchi Optimization Method

**Figure 8:**
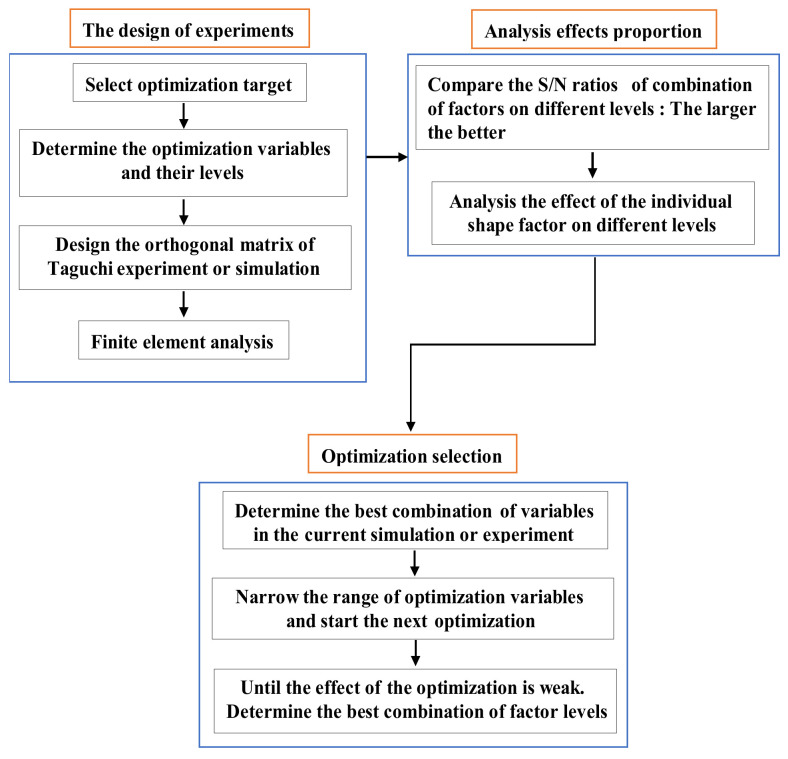
The flowchart of the Taguchi optimization method.

The Taguchi optimization algorithm, which is adept in the use of discrete results to identify the optimal point, has been widely used in many areas, e.g., national defense, chemical, and motor applications [23]. The orthogonal array (OA) and the signal-to-noise(S/N) ratio are the main tools used in this method [24,25,26]. The former was applied to design representative experiments and shorten the number of required hours. The latter was used to analyze the factors’ effect and determine the next optimization. Figure 8 shows the flowchart of the Taguchi optimization method. 

The THD-min objective functions for the 3P-HSM can be expressed by

(7) $$\mathrm{THD}‐\min=\min\left(\sqrt{\sum_{n=2}^{\infty }V_{n}^{2}}/V_{1}\right)$$

where *V*_1_ is the fundamental component of output voltage and *V_n_* represents other harmonic components.

Using the Taguchi method, three formulas can be applied to calculate the S/N ratio:

(8) $$SN_{N}\;=\;10\log\left({\bar{y}}^{2}/s^{2}\right)$$

(9) $$SN_{L}\;=\;-10\log\;\left[\left(\sum_{i=1}^{n}1/y_{i}^{2}\right)/n\right]$$

(10) $$SN_{S}\;=\;-10\log\left[\left(\sum_{i=1}^{n}y_{i}^{2}/n\right)\right]$$

where *ȳ* is the average value of objective functions, *s* is the standard deviation, *n* represents the repeat numbers in each test and *y_i_* is the output of the test in the *i*^th^ repetition.

Equation (10) was adopted in the optimization target (THD-min), but the value of the S/N ratio needed to be maximal.

### 4.2. The Optimization Target: THD-min

In HSMs, the torque-angle characteristic is generally not sinusoidal and it is evidently affected by harmonics, especially the second ones, which will reduce motor performance and increase motor noise [27,28]. In this study, it was desired to optimize the tooth geometry in order to reduce the harmonic.

In this paper, the HSM with the original structure is defined as “Initial 3P-HSM”, and the motor with the optimized structure is denoted as “THD-min”. The optimization process of THD-min 3P-HSM was divided into two stages. The optimization parameters of each stage are shown in Table 3, and the formulations of S/N adopted in this section are shown as Equations (7) and (10).

**Figure 9:**
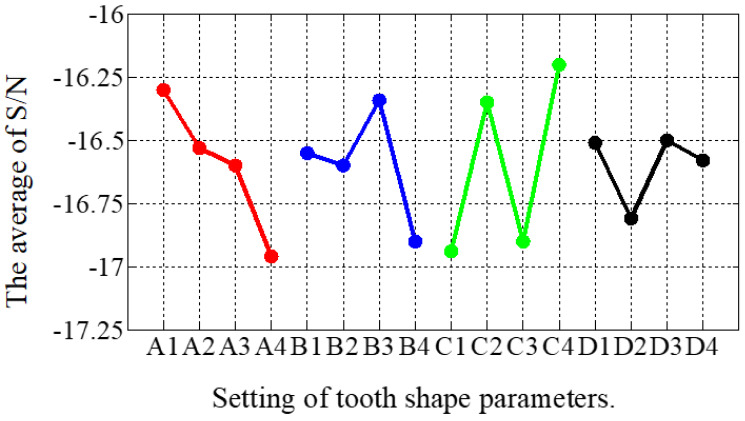
The main effect diagram of S/N for THD-min in the first stage.

The combinations of signal-to-noise ratios, at different levels, for the four factors (A = r_t1_/r_λ_, B = s_t1_/s_λ_, C = r_θ_, D = s_θ_) during the optimization process are shown in Table 4. The effect of each shape factor is reflected in Figure 9. It is clear that except that factor D had the least impact, the effect of each tooth shape factor was not significantly different. According to the average value of the S/N ratio, the best factor level combination can be judged as A1B3C4D3, where A1 = 0.15, B3 = 0.1, C4 = 15, and D3 = 60. After the first stage optimization, the THD of the THD-min 3P-HSM was reduced from 7.55% to 6.2% of the prototype’s initial 3P-HSM.

**Figure 10:**
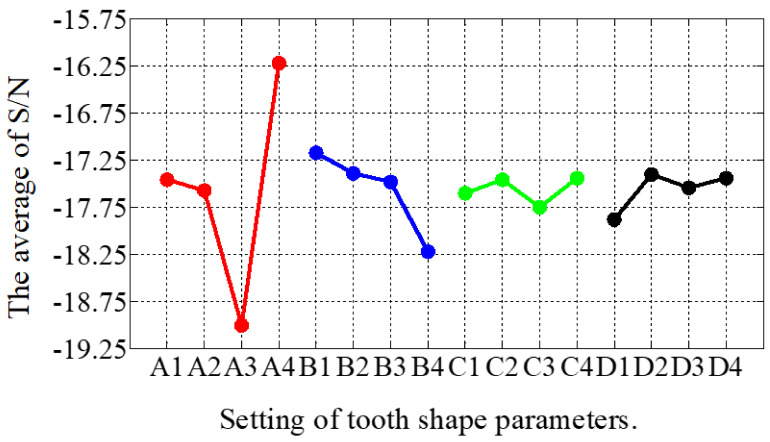
The main effect diagram of S/N for THD-min in the second stage.

In the second stage of optimization, the impact of the parameters from the first stage was analyzed and the parameters for the new optimization were adjusted, as shown in Table 3. From Table 5 and Figure 10, the simulation results and the influence of the individual shape factors for the second stage, respectively, can be found. It is clear that A played a decisive role in the THD values, which were generally smaller when A was at level 4. The best combination of factor levels for the second stage was A4B1C4D2, where A4 = 0.45, B1 = 0.55, C4 = 0.2, and D2 = 0.6. Furthermore, the THD for the best combination of factor levels decreased from 6.2% to 5.96% in the first stage. Clearly, the second stage was relatively weak and, thus, the optimization was over at this stage.

### 4.3. Tooth Geometry after Optimization and FE Results

As the EM structure of HSM is very complicated, the traditional equivalent magnetic circuit method and other analytical methods could not accurately calculate its torque, and thus, a 3D FE model had to be used to calculate the torque-angle characteristics of the HSMs.

In the optimization process, discrete data on the magnetic potential of the motors were obtained with FEM, which was then used to calculate the EM torque of the motor. In the calculation, the torque was obtained using the energy method. This was due to the fact that the energy method was able to use the global results of the FE to calculate the torque with a high degree of accuracy. The formula for calculating the EM torque using the energy method is as follows:

(11) $$T=\frac{dW(\theta ,i)}{d\theta }$$

where *W(θ,i)* is the magnetic common energy and *i* is the excitation winding current.

**Figure 11:**
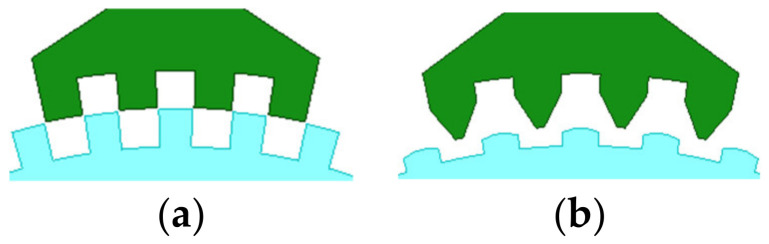
Tooth geometry after optimization: (**a**) initial 3P-HSM; (**b**) THD-min 3P-HSM.

**Figure 12:**
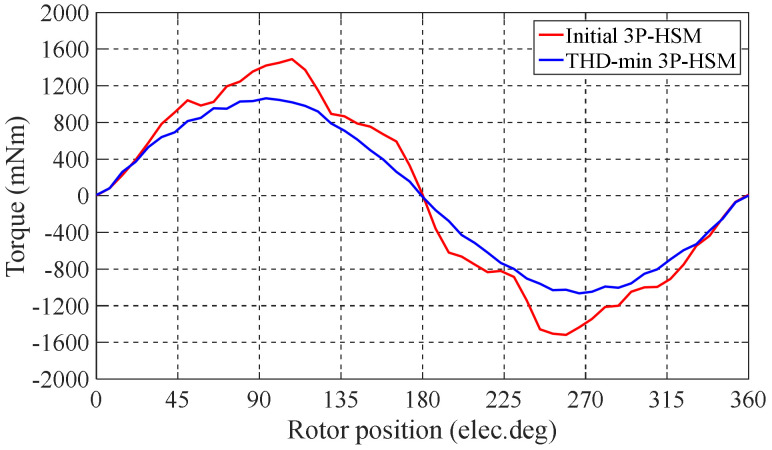
The FE results of 3P-HSMs.

The optimal tooth geometry and the FE results of the 3P-HSMs are illustrated in Figure 11. Furthermore, the FE analysis results of the torque angle characteristic are shown in Figure 12. It is clear that the THD of the optimized HSM was significantly improved compared to the initial design.

Table 6 shows that the THD of the optimized 3P-HSM was 5.96%, which was 21% less than the 7.55% value of the initial 3P-HSM. The reduction in THD was beneficial in terms of reducing the torque ripple in the steady state operation of the motor in order to reduce the operation noise, which was verified by the test results that are included in the coming section. From Table 5, it can be seen that only 16 samples were used in the optimization, and the number of calculations using FEM was significantly reduced.

## 5. Motor Noise Test

For a 3P-HSM, due to its unique multi antipole structure, the high-order harmonic torque ripples are difficult to measure directly. However, the motor noise caused by torque ripple can be obtained by means of a noise test, which can be used to analyze the torque ripple of the motor [29,30]. 

**Figure 13:**
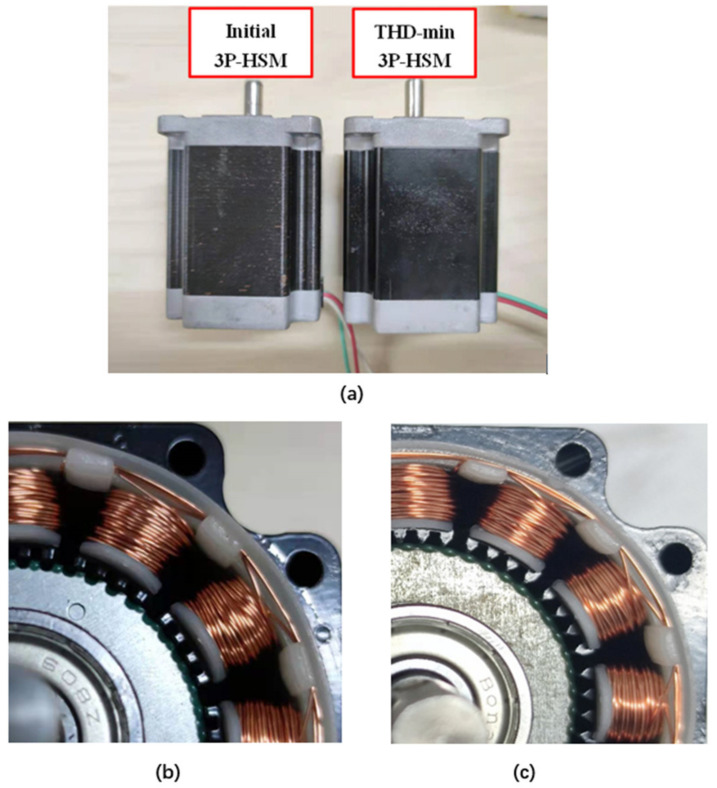
Test prototypes and tooth layer: (**a**) test prototype; (**b**) rectangular; (**c**) trapezoidal.

To verify the optimization, in addition to “Initial 3P-HSM”, which is a commercial product, “THD-min” was also prototyped and used in the test. These two stepper motors are shown in Figure 13. 

Noise testing of motors involves high requirements for the testing environment; thus, the motor noise tests carried out in this research were conducted in an acoustic chamber [30]. The chamber used multiple layers of grille fabric and multiple layers of damped acoustic composites to absorb the noise and prevent multiple sound reflections from occurring. A special damping spring was installed at the bottom of the acoustic chamber to prevent external ambient noise from entering the acoustic chamber and causing interference [31,32,33]. Table 7 shows the equipment used in the noise tests. In the test, the motor and microphone were installed in the acoustic chamber for noise measurement. The host computer drove the 3P-HSM with a sinusoidal current, and recorded motor noise information at different speeds. Then, waterfall analysis was used for vibration and noise analysis, which superimposed each speed in a graph to display the noise spectrum. 

**Figure 14:**
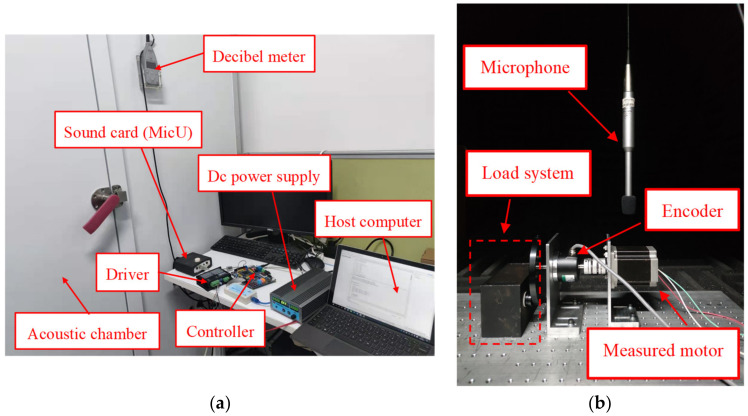
The experimental noise system: (**a**) outside of the testing system; (**b**) inside of the acoustic chamber.

The experimental system is shown in Figure 14.

**Figure 15:**
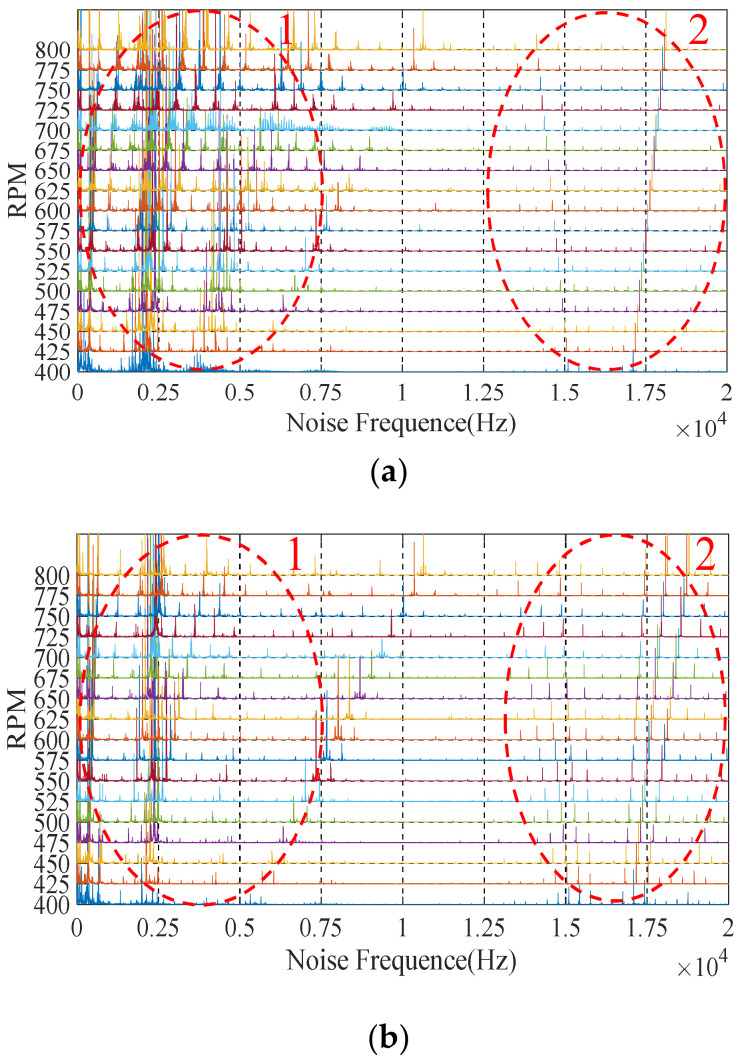
Complete noise waterfall: (**a**) initial 3P-HSM; (**b**) THD-min 3P-HSM.

During the test, the noise information of the motor was collected by the host computer and the Fast Fourier Transform (FFT) analysis was used to obtain the noise spectrum of the motor. Figure 15 shows the complete noise spectrum under the sinusoidal drive current of 3P-HSM, including the low-frequency harmonic part (ellipse-1) and the chopping frequency part (ellipse-2).

From Figure 15, it can be found that the noise amplitude of THD-min 3P-HSM was significantly reduced, which shows that the reduction in THD caused a significant improvement in motor noise. Furthermore, the noises near the ordinate axis were chaotic, which was related to the working environment and the internal friction of 3P-HSM and will not be discussed here. 

**Figure 16:**
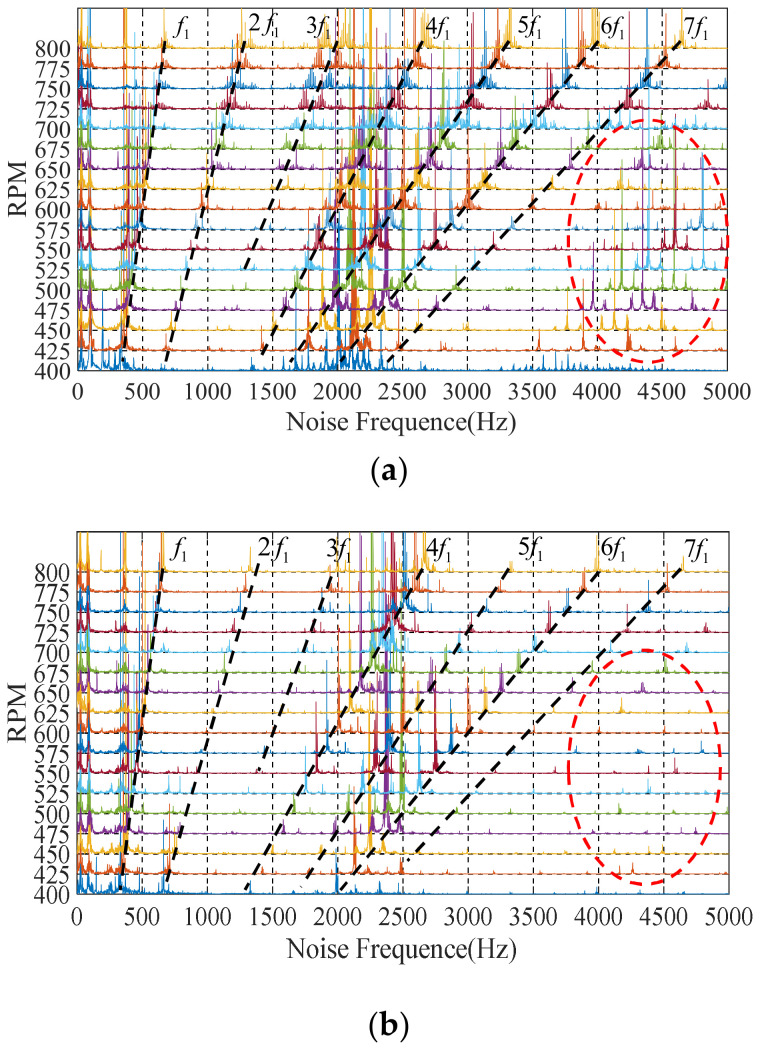
Harmonic noise waterfall: (**a**) initial 3P-HSM; (**b**) THD-min 3P-HSM.

The noise frequency linked with the motor drive current is:

(12) $$f_{i}=i\times p\times \frac{n}{60}$$

where *f_i_* is the *i*^th^ order harmonic frequency component of the drive current, *n* is the motor’s synchronous speed, and *p* is the number of pole pairs of 3P-HSM. Therefore, the fundamental frequency of the drive current generating EM noise can be calculated

(13) $$f_{1}=1\times 50\times \frac{400}{60}=333\mathrm{Hz}$$

and this is represented by the dotted line *f_1_* in Figure 16. Moreover, due to the existence of harmonic EM torque, the 3P-HSM would also generate noise at the integral multiple harmonics of the fundamental frequency.

Compared with the initial prototype, the noise amplitude of THD-min 3P-HSM was significantly reduced, especially the reduction in the harmonic noise by 6 times, as well as the reduction in the higher harmonics, indicating that the torque ripple was improved in the optimized motor; this means that the optimization of the tooth shape was effective.

Additionally, in Figure 15, the noise at 15–20 kHz is shown to be caused by the chopping of the driver. Because the chopping frequency of the current was 16.2 KHz, the noise formed by the chopping was distributed in an umbrella shape centered at 16.2 KHz on the waterfall diagram. It appeared as a result of the modulation of the high frequency carrier signal and the low frequency modulating wave signal and was not related to the tooth shape of the motor; thus, it is not discussed in detail here.

**Figure 17:**
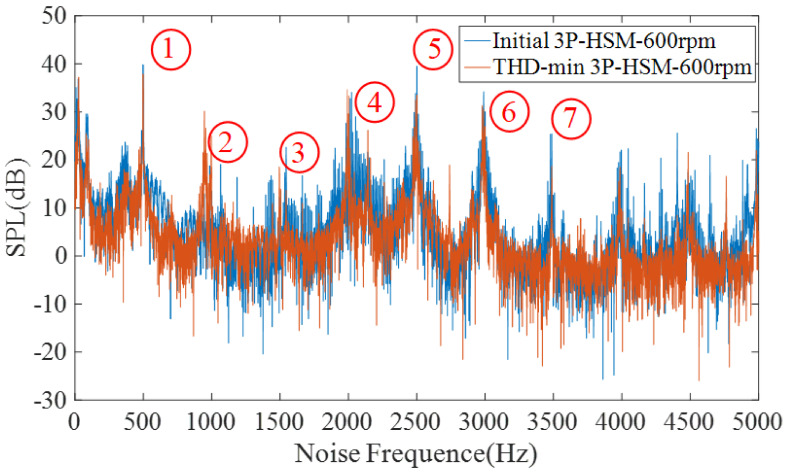
SPL spectrum at 600 rpm.

Figure 17 shows the sound pressure level (SPL) spectrum of 3P-HSM operating at 600 rpm. The noise levels of the two shapes can thus be compared in Table 8. 

As shown in Figure 17, the SPL amplitude of decreased with the optimization of the motor tooth layer structure, and the motor noise was suppressed. The data in Table 8 also reveal that the noise value of THD-min 3P-HSM at different harmonic frequencies was smaller than that of the initial 3P-HSM. Furthermore, from 3500 Hz to 5000 Hz, the noise fluctuation of THD-min 3P-HSM was evidently decreased. All of these indicate that the new trapezoidal tooth shape is effective in reducing the torque ripple and improving the performance of 3P-HSM.

## 6. Conclusions

The “torque-angle characteristic” of a hybrid stepper motor is closely related to the tooth shape of the motor. Optimization of the tooth shape of such motors is difficult because of the complexity of the motor structure, the small air gap of the motor, and the presence of both axial and radial magnetic fields. The three-dimensional finite element method is an effective tool for the analysis of such motors, but they are extremely computationally intensive and require very long computation times, making them ineffective for the optimization of hybrid stepper motors. In order to solve the problem of computational effort, an optimization method combining the FEM and Taguchi method is presented in this paper. The method was found to be able to make full use of the information from the FEM results to analyze and determine the direction of optimization with fewer discrete FE results, thus greatly reducing the number of times FEM needs to be undertaken in the optimization process and allowing the optimization to be implemented. This paper presents the results obtained using this method to optimize the tooth shape of a three-phase hybrid stepper motor with the objective of reducing the THD of the “torque-angle characteristic”. The performances of the motors with the original design and optimized design were analyzed. The new trapezoidal tooth shape reduced the THD of the “torque-angle characteristic” of the motor by 21% compared to the conventional rectangular tooth shape, which resulted in a significant improvement in motor noise. Therefore, the “FEM + Taguchi method” proposed in this paper is effective and can be used for the optimization of three-phase hybrid stepper motors, as well as for other types of motors.

## Figures and Tables

**Table 1:** Main parameters of HSMs.

Parameters	Values
Stator poles	12
Small teeth stator pole	4
Rotor layers	4
Small teeth per rotor layer	50
Permanent magnets	3

**Table 2:** Geography of teeth for 3P-HSMs.

Model	Tooth Geometry
Initial 3P-HSM	Rectangular
THD-min 3P-HSM	Trapezoidal

**Table 3:** Optimization parameters of THD-min.

Optimization Parameters	Optimization Stage	
	First Stage	Second Stage
A	r_t1_/r_λ_	r_t_/r_λ_
B	s_t1_/s_λ_	s_t_/s_λ_
C	r_θ_	r_h_/r_λ_
D	s_θ_	s_h_/s_λ_

**Table 4:** The simulation results of THD-min 3P-HSM in the first stage.

No.	Factor				THD (%)	S/N
	A	B	C	D		
1	1	1	1	1	7.36	−17.34
2	1	2	2	2	7.10	−17.03
3	1	3	3	3	7.50	−17.50
4	1	4	4	4	7.91	−17.96
5	2	1	2	3	7.20	−17.15
6	2	2	1	4	7.30	−17.27
7	2	3	4	1	7.68	−17.71
8	2	4	3	2	8.10	−18.17
9	3	1	3	4	8.60	−18.69
10	3	2	4	3	8.51	−18.60
11	3	3	1	2	8.80	−18.89
12	3	4	2	1	9.81	−19.83
13	4	1	4	2	5.96	−15.50
14	4	2	3	1	6.80	−16.65
15	4	3	2	4	6.19	−15.83
16	4	4	1	3	7.00	−16.90

**Table 5:** The simulation results of THD-min 3P-HSM in the second stage.

No.	Factor				THD (%)	S/N
	A	B	C	D		
1	1	1	1	1	6.54	−16.31
2	1	2	2	2	6.56	−16.34
3	1	3	3	3	6.20	−15.85
4	1	4	4	4	6.84	−16.70
5	2	1	2	3	6.80	−16.65
6	2	2	1	4	6.66	−16.47
7	2	3	4	1	6.20	−15.85
8	2	4	3	2	7.20	−17.15
9	3	1	3	4	7.00	−16.90
10	3	2	4	3	6.25	−15.92
11	3	3	1	2	7.43	−17.42
12	3	4	2	1	6.44	−16.18
13	4	1	4	2	6.56	−16.34
14	4	2	3	1	7.66	−17.68
15	4	3	2	4	6.48	−16.23
16	4	4	1	3	7.56	−17.57

**Table 6:** Tooth shape parameters of two prototypes.

Parameters	Initial 3P-HSM	THD-min 3P-HSM
r_t1_	0	0.35
s_t1_	0	0.4
r_θ_	0	25
s_θ_	0	35
r_t_	0.45	0.5
s_t_	0.5	0.5
r_h_	0.5	0.4
s_h_	0.5	0.5
THD (%)	7.55	5.96

**Table 7:** Test equipment types.

Test Equipment	Company	Type
Controller	TI	DSPF28335
Sound card	iCON	MicU(Live)
Microphone	Behringer	ECM8000
Decibel meter	Aihua	AWA5661

**Table 8:** The value of SPL spectrum at different harmonic points.

Tooth Geometry	SPL Spectrum(dB)						
	1	2	3	4	5	6	7
Rectangular	30.75	21.18	12.81	19.73	41.92	33.69	29.25
Trapezoidal	29.1	10.12	7.443	12.95	36.72	30.33	17.48

## Data Availability

Not applicable.
